# Metabolic parameters of sequential 18F-FDG PET/CT predict overall survival of esophageal cancer patients treated with (chemo-) radiation

**DOI:** 10.1186/s13014-019-1236-x

**Published:** 2019-02-19

**Authors:** Yimin Li, Sebastian Zschaeck, Qin Lin, Sijia Chen, Lili Chen, Hua Wu

**Affiliations:** 1 Department of Radiation Oncology, Xiamen Cancer Hospital, the First Affiliated Hospital of Xiamen University, Xiamen, China; 2 Department of Radiation Oncology, Charité Universitätsmedizin Berlin, Berlin, Germany; 3 Department of Nuclear Medicine, Xiamen Cancer Hospital, the First Affiliated Hospital of Xiamen University/Southern Fujian PET Center, Xiamen, China

**Keywords:** Esophageal cancer, 18F-FDG PET/CT, Chemoradiotherapy

## Abstract

**Background:**

To evaluate the prognostic value of metabolic parameters of pre-treatment and interim 18F-fluorodeoxyglucose (FDG) positron emission tomography (PET) for overall survival (OS) of esophageal cancer(EC) patients undergoing (chemo-) radiotherapy.

**Methods:**

A retrospective analysis of 134 patients with pathology confirmed squamous cell EC treated between July 2009 and October 2013 in our hospital was performed. Inclusion criteria for this study were curative intended radiotherapy and availability of pre-treatment and interim 18F-FDG PET. 18F-FDG PET/CT scans were acquired before treatment and after 40 Gray (Gy) of radiotherapy. Maximum standard uptake value (SUV_max_), metabolic tumor volume(MTV), total lesion glycolysis (TLG), and the percentual changes during both PET scans were recorded. The parameters were named as SUVmax1,MTV1,TLG1,SUVmax2,MTV2,TLG2,△SUV_max_,△MTV and △TLG. The receiver operating characteristic curve (ROC) was used to analyze the relationship between metabolic parameters and OS, survival analysis was carried out by Kaplan-Meier and Cox regression analysis.

**Results:**

Univariate survival analysis showed that SUVmax2、MTV1、△MTV、TLG1、TLG2 and △TLG were associated with OS. Based on the largest Youden index of ROC curves, patients with SUVmax2 < 7.8, MTV1 < 10.5, △MTV < 0.075, TLG1 < 59.8, TLG2 < 44.3 and △TLG < 0.27 tended to live longer. Stratified for these parameters, the estimated median survival time were 27.9 months (m) vs 9.8 m, 36.9 m vs 11.3 m, 41.6 m vs 12 m, 48.9 m vs 14.3 m, 32.6 m vs 13.2 m, and 41.6 m vs 14.5 m. Cox multi-factor regression analyses revealed SUV_max_2 as an independent prognostic factor for OS complementary to TNM staging and the length of primary tumor.

**Conclusions:**

Sequential 18F-FDG PET/CT metabolic parameters bear important prognostic value for OS of EC patients. 18F-FDG PET/CT scan before treatment and during chemoradiotherapy seems helpful to evaluate the effect of chemoradiotherapy, guide clinical decisions and provide patients with personalized treatment.

## Background

Esophageal cancer (EC) is the eighth most common cancer worldwide with the sixth highest mortality [1]. In China, EC incidence is high, accounting for 50% of all new global EC cases annually and most of them present with advanced stage. Patients with early stage EC can be treated by surgery alone, while those with locally advanced EC usually receive radiotherapy combined with platinum-based chemotherapy [2]. Neo-adjuvant chemoradiotherapy before surgery is also a common treatment regime [3, 4]. The optimal primary treatment method is controversial. Both definitive radiochemotherapy (RCT) and preoperative RCT followed by radical surgery are comparable regarding overall survival (OS) of patients; however, conclusive clinical data from state of the art phase III studies are lacking. Moreover, some recent analyses suggest better OS after trimodality treatment [5–8]. Since local recurrences appear to occur more frequently after definitive RCT, trimodality treatment is usually the treatment of choice in medically fit patients; however, in some patients, the location (mostly cervical) or extent of the primary tumour impedes radical tumour excision, or the patient refuses to undergo surgery.

At present, EC treatment outcomes are usually evaluated by computed tomography (CT) and a barium swallow test, but these examinations provide only anatomical information and very coarse functional information. 18F-FDG PET/CT is a functional imaging modality that can predict EC prognosis more accurately than conventional CT [9, 10].As biological status of tumors is evolving and metabolic tumor response can be detected earlier than morphological tumor response by CT, sequential 18F-FDG PET/CT seems to be an especially useful prognostic/predictive tool. Some previous studies showed that sequential 18F-FDG PET/CT during treatment is able to predict outcomes after radiotherapy and chemotherapy for EC [11].The commonly used metabolic parameters for predicting EC patients’ survival include maximum of standard uptake value (SUV_max_) and metabolic tumor volume (MTV).The prognostic value of SUV_max_ remains inconclusive [12–15], and the prognostic value of MTV is affected by esophageal radiation inflammation during radiotherapy [16–18].Total lesion glycolysis (TLG),the product of the MTV and mean SUV, has emerged as a relatively new 18F-FDG PET/CT prognostic parameter in recent years. Studies have shown that TLG is more reliable than MTV because TLG reflects both mean metabolic FDG uptake and tumor volume. Currently, TLG was suggested to predict the treatment outcome of neoadjuvant therapy in smaller number of patients treated in other countries [19, 20], only few studies have been conducted in China to investigate the value of TLG in predicting(chemo-)radiation outcomes for EC.

In our study, 18F-FDG PET/CT was performed before treatment and during radiotherapy in patients with esophageal squamous cell carcinoma undergoing (chemo-)radiation. The sequential 18F-FDG PET/CT metabolic parameters were collected. The aim of our study is to find out whether the metabolic parameters and the change rate of them have prognostic value in Chinese patients.

## Methods

### General information

We retrospectively analyzed clinical and PET data from EC patients who received radiotherapy and underwent 18F-FDG PET/CT before and during treatment at our hospital between July 2009 and October 2013. Some of the patient data has already been published with focus on other parameters, this is an additional analysis with long-term follow up and the following inclusion criteria: pathology proven esophageal cancer, age > 18 years, I-IVa disease at staging, initial treatment of esophageal cancer, absence of distant metastasis or other concomitant tumors, no contraindication to radiotherapy or chemotherapy; ECOG score: 0~2, Karnofsky performance status(KPS) score ≥ 70; no major organ dysfunction, no serious anomalies of blood routine test, liver function, pulmonary function, renal function and cardiac function; assay indexes requirement: WBC ≥ 4.0 × 109 /L, ANC ≥ 1.5 × 109 /L, PLT ≥ 100 × 109 /L, Hb ≥ 90 g/L; serum bilirubin≤1.5 times of normal high limit, AST, ALT≤1.5 times of normal high limit, creatinine≤normal high limit, normal ECG; 18F-FDG PET/CT before treatment and interim PET at 4–5 weeks during radiotherapy with complete imaging data available.

Clinical staging was determined according to the *Non-Surgical Treatment of Esophageal Cancer Clinical Staging Criteria* (draft) from the Fifth National Symposium on Radiotherapy for Esophageal Cancer(2010) [21]. Each patient provided written informed consent. A total of 134 EC patients were enrolled, and the clinical features are shown in Table 1.

**Table 1:** Patient characteristics

Clinical Features	n (%)
Age (y)	
62.4 ± 9	–
Range (42–88)	–
Sex	
F	32 (23.9%)
M	102 (76.1%)
Grade of differentiation	
Highly differentiated (G1)	12 (9.0%)
Moderately differentiated(G2)	95 (70.9%)
Poorly differentiated(G3)	22 (16.4%)
Undifferentiated (G4)	5 (3.7%)
Primary tumor site	
Cervical	13 (9.7%)
Upper thorax	44 (32.8%)
Middle thorax	60 (44.8%)
Lower thorax	14 (10.4%)
Mixed	3 (2.2%)
Length of primary tumor	
< 3.5	27(20.2%)
> 3.5	104 (77.6%)
Not available	3 (2.2%)
*T stage	
T1	3 (2.2%)
T2	8 (6.0%)
T3	43 (32.1%)
T4	80 (59.7%)
*N stage	
N0	49 (36.6%)
N1	50 (37.3%)
N2	35 (26.1%)
Concurrent chemotherapy	
No	48 (35.8%)
Yes	86 (64.2%)

*TNM staging was determined based on the Clinical non operative treatment of esophageal cancer staging criteria (draft, 2010)

### Radiotherapy

Patients were treated with conventional fractionated RCT with a single dose of 1.8 or 2 Gy per fraction. From high-resolution contrast-enhanced CT and 18F-FDG PET, gross tumour volume (GTV) was contoured separately for the primary tumour and affected lymph nodes. The clinical target volume (CTV) of the primary tumour was generated by enlarging the primary GTV by 4 cm in craniocaudal extension of esophageal lesion and by 0.5 cm in radial extension. Additionally, regional lymph node regions were included with sufficient safety margins within the nodal CTV, and all CTVs were adapted to anatomical structures (excluding bones, lungs or large vessels). The planning treatment volume (PTV) comprised the CTV with additional margins of 0.5 cm. After administration of 50 Gy, an additional radiation boost of 4–16 Gy (average 58.9 Gy) was prescribed to a reduced treatment volume including only the GTV with reduced safety margins (PTV boost).

### Chemotherapy

Patients with stage T3 and/or N+ received concurrent cisplatin-based chemotherapy starting on the day before radiotherapy. Chemotherapy regimens were as follows:

- Cisplatin 25 mg/m^2^, on days (d) 1–3
- Paclitaxel 135 mg/m^2^(d1) or5-FU 500 mg/m^2^, continuous on 5 days (d1-d5)
- Two cycles were given concomitant to radiotherapy with 28 days between cycles

### 18F-FDG pet/CT

18F-FDG PET/CT was performed at the following time points: 1)within 4 weeks before start of radiotherapy and chemotherapy (whole-body imaging) and 2)when 40–50 Gy to the PTV had been delivered (chest imaging, including all initial EC lesions). Metabolic parameters SUVmax, MTV, and TLG were measured. SUV_max_1, MTV1, and TLG1 indicates pretreatment values; SUV_max_2, MTV2, and TLG2 indicates values obtained during interim PET; △SUV_max_, △MTV, and △TLG indicates the change (%): △SUV_max_ = (SUV_max_1-SUV_max_2)/SUV_max_1, △MTV = (MTV1-MTV2)/MTV1, and △TLG = (TLG1-TLG2)/TLG1.

FDG PET/CT image acquisition was performed on a Discovery STE (GE Medical Systems, Milwaukee, WI, USA) with standard settings. 18F-FDG (0.15 mCi/kg) was generated using a cyclotron (Discovery STE; GE Medical Systems, Milwaukee, WI, USA) at our hospital. After injection, the patient rested for 50–60 min before the acquisition started and a 16-slice spiral CT scan was performed at 140 kV, 120mAs, 3.75 mm thickness, and a 3.75 mm interval for attenuation correction calculation and anatomical localization.

Imaging data were interpreted by at least two experienced nuclear medicine physicians and, with reference to clinical signs and symptoms, gastroscopy, barium swallow testing, and CT images, were used to select the region of interest (ROI) in the esophageal lesions and the localized high radioactivity areas in the cervical, thorax, and abdominal areas at the Xeleris Workstation (GE Healthcare, Version 4.3). The selected ROI was manually adjusted to exclude physiologically high uptake areas such as the heart, and 40% of SUV_max_ was used as the lower threshold for MTV calculations. SUV_max_, mean SUV (mean of standard uptake value, SUV_mean_), MTV, and TLG were recorded during both 18F-FDG PET/CT scans. The parameters were calculated as follows: SUV = radioactivity of the sensitive area/ratio of the injected dose to the patient’s weight, SUV_max_ was the maximum SUV in the ROI, MTV was the volume included in the curve greater than or equal to 40% SUV_max_, SUV_mean_ was the mean SUV in the MTV, and TLG was calculated as SUV_mean_ × MTV. If patients did not have complete remission during (chemo-)radiotherapy, both PET scans would be co-registered and the parameters were determined based on the primary lesion’s original location identified by visual method.

### Follow-up

Patients were followed up from the start of treatment to the end of the follow-up period. Data from patients who died of any reasons were classified as complete data; data from patients who achieved tumor-free survival, or survival with tumor were censored at the date of the last follow-up information. Survival was defined as the time from the start of treatment to death or the end of follow-up. Barium swallow tests and chest 18F-FDG PET/CT scan were performed one month after radiotherapy, then barium swallow tests and chest CT were performed every three months during the first 2 years and every six months for the next 3 years. After 5 years of treatment, barium swallow tests and chest CT scans were carried out once a year. Recurrence or metastasis needed to be confirmed by continuous imaging or biopsy.

### Statistical analysis

Measurement data were expressed as the mean ± standard deviation (x ± s).SPSS Statics 22 (IBM, Armonk, NY) was used for all statistical analysis. Statistically significant parameters were analyzed using the operator characteristic curve (ROC) to obtain the optimal threshold based on the Youden index. The associations between endpoints and clinically relevant parameters (gender, age, tumour grade, T stage, N stage, UICC stage and localization) as well as quantitative PET parameters were analyzed using univariate Cox proportional hazards regression in which the PET parameters were included as binarized parameters. The Kaplan-Meier method (log-rank test) and Cox proportional hazards regression models were used to analyze OS and associated risk factors. Values ≤ α = 0.05 were deemed statistically significant.

## Results

### Clinical features, follow-up, and survival

A total of 134 patients were eligible and included in this analysis. Patients were treated between July 2009 and October 2013. Follow up ended on October 1, 2017, with a median follow-up time of 62(47–99) months. 101 patients died and 33 patients survived during follow-up. The 1-, 3-, and 5-year overall survival rates were 66.4, 35.7, and 24.5%, respectively. The 1-, 3-, and 5-year local control rates were 73.3, 57.5, and 54.5%, respectively, and the 1-, 3-, and 5-year progress free rates were 48.5, 24.4, and 21.3%, respectively.

### Relationships between each baseline 18F-FDG PET/CT parameters and OS

**Fig. 1:**
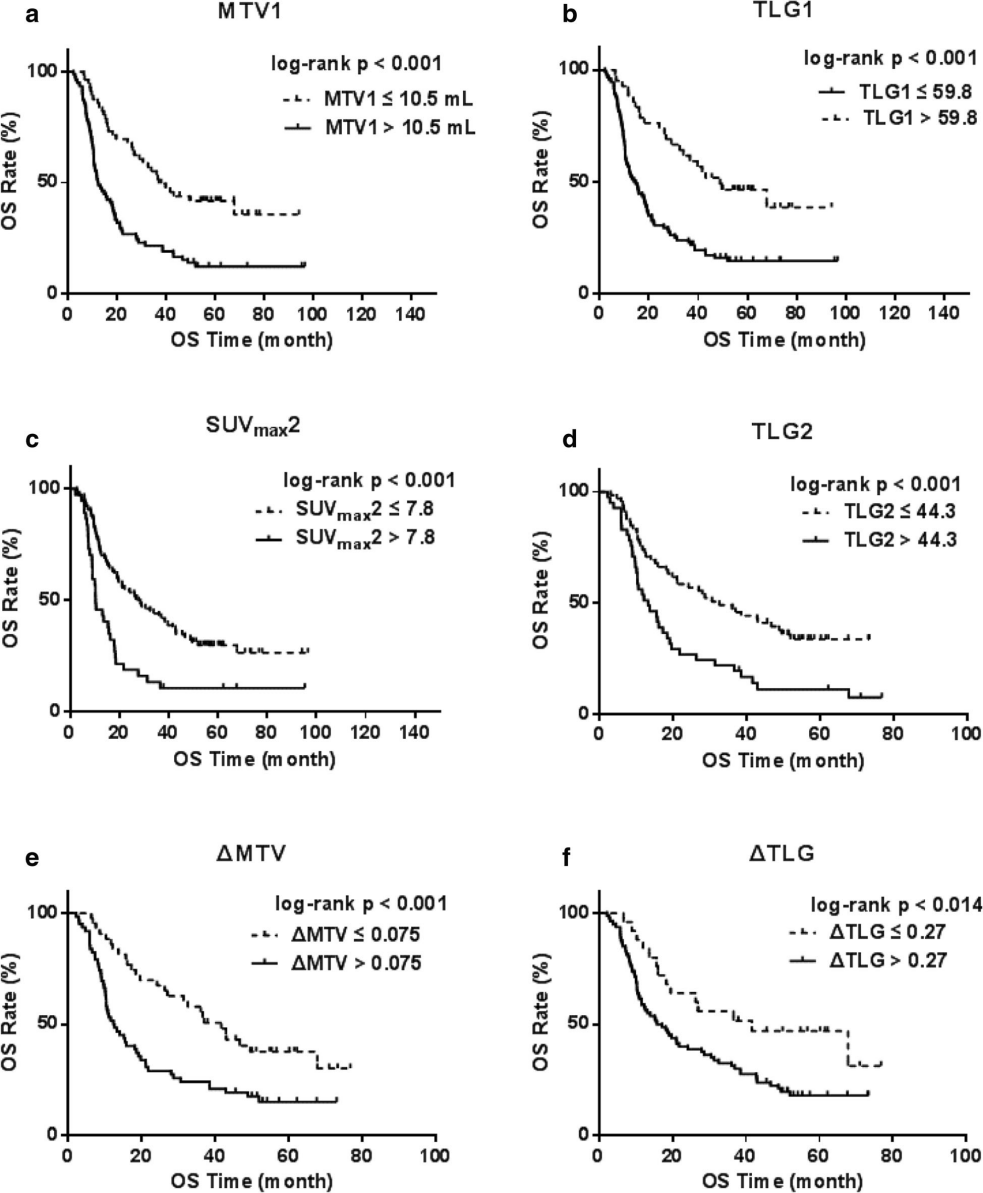
Kaplan-Meier curves showing the relationship between different 18F-FDG PET/CT metabolic parameter and OS; **a**. Relationship between MTV1 and OS **b**. Relationship between TLG1 and OS. **c**. Relationship between SUV_max_2 and OS **d**. Relationship between TLG12and OS;**e**. Relationship between ΔMTV and OS **f**. Relationship between ΔTLG and OS

Mean SUV_max_1 was 13.9 ± 6.0, mean MTV1 was 18.8 ± 18.8 mL, and mean TLG1 was 159.4 ± 154.0. Univariate survival analysis showed that SUV_max_1 was not associated with OS (Table 2). As shown in Table 2 MTV1 and TLG1 were both associated with OS. The optimal prognostic threshold for OS (per the ROC and Youden index) were 10.5 mL for MTV1 and 59.8 for TLG1. Figure 1a and Fig. 1b show Kaplan-Meier plots for both parameters.. Log-Rank test of Kaplan-Meier survival analysis showed that MTV1 < 10.5 mL and TLG1 < 59.8 were associated with improved OS, with an estimated median survival of 36.9 months (95% CI: 26.4–47.5, *p* < 0.0001) and 48.9 months (95% CI: 25–72.7, p < 0.0001), respectively.

**Table 2:** Univariate Cox regression analysis with respect to overall survival

Parameter	Risk	HR	95% CI	*p* value
Clinical parameters				
Gender	Male	1.18	0.74–1.89	0.477
Age	> 58.5	1	0.98–1.02	0.698
Tumor_grade	> 2	2.63	1.71–4.05	**0**
T stage	> 3	1.52	1.10–2.08	**0.01**
N stage	> 1	1.46	1.13–1.89	**0.003**
UICC stage	>II	2.19	1.50–3.22	**0**
Localization	middle	1.31	1.04–1.65	**0.02**
Length of tumor	> 3.5	1.18	1.08–1.28	**0**
Concurrent chemo	No	0.99	0.66–1.48	0.943
PET parameter				
SUV_max_1	> 9.6	1.01	0.98–1.04	0.419
SUV_max_2	> 7.8	1.16	1.07–1.25	**0**
ΔSUV_max_	< 0.23	0.85	0.51–1.42	0.538
MTV1	> 10.5	1.02	1.01–1.03	**0**
MTV2	> 15.9	1.01	0.99–1.03	0.395
ΔMTV	> 0.075	1.17	0.98–1.40	**0.001**
TLG1	> 59.8	1	1.0–1.01	**0.001**
TLG2	> 44.3	1.01	1.0–1.01	**0.019**
ΔTLG	> 0.27	1.21	0.91–1.62	0.014

*HR* Hazard ratio, *CI* Confidence interval

Values in bold are showing at least trend for significance with *p* < 0.1 in univariate analyses

### Relationships between interim 18F-FDG PET/CT parameters and OS

Performed after application of 40 Gy, the mean SUV_max_2 was 6.42 ± 2.7, the mean MTV2 was 13.9 ± 11.0 mL, and the mean TLG2 was 45.6 ± 30.8. Univariate survival analysis showed that MTV2 was not significantly associated with OS, while SUV_max_2 and TLG2 were significantly related to OS (Table 2).As show in Fig. 1c and Fig. 1d, the optimal prognostic threshold for OS was 7.8 for SUV_max_2 and 44.3 for TLG2. Log-Rank test of Kaplan-Meier survival analysis showed that SUV_max_2 < 7.8 and TLG2 < 44.3 were associated with improved OS, with an estimated median survival of 27.9 months (*p* < 0.001) and 32.6 months (*p* < 0.001), respectively.

### Relationships between changes in 18F-FDG PET/CT metabolic parameters and OS

Mean ΔSUV_max_ was 0.46 ± 0.34, mean ΔMTV was − 0.25 ± 1.42, and mean ΔTLG was 0.38 ± 0.92.Univariate survival analysis showed that ΔSUV_max_ was not significantly associated with OS, while ΔMTV and ΔTLG both showed a significant association with OS (Table 2). The optimal prognostic thresholds for OS (per the ROC and Youden index) were 0.075 and 0.27, respectively. Kaplan-Meier survival analysis showed that ΔMTV < 0.075 and ΔTLG < 0.27 were associated with improved OS, with an estimated median survival of 41.6 months for both parameters (p < 0.001, *p* < 0.014) (Fig. 1e and Fig. 1f).

### Relationships between clinical features and OS

**Fig. 2:**
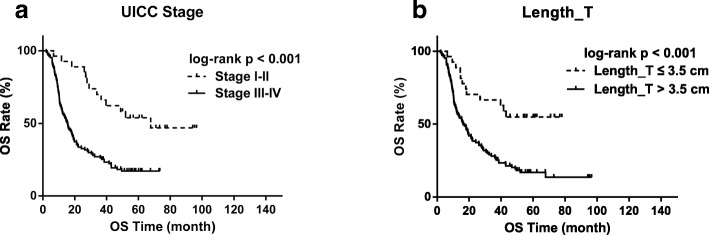
Kaplan-Meier curves for overall survival (OS) in patients stratified by UICC stage and length of primary tumor

Univariate survival analysis showed that UICC stage and lesion length were significantly associated with OS. ROC analysis showed that Stage I-II and a lesion length (Length_T) ≤ 3.5 cm was associated with improved median survival, which was 67.8 months (*p* < 0.001) in stage I-II patients and was not reached in the group with a tumor length_T ≤ 3.5 cm (p < 0.001), respectively (Fig. 2).

### Multivariate analysis

UICC stage, Length_T, SUV_max_2, MTV1, ΔMTV, TLG1, TLG2, and ΔTLG were incorporated into Cox multivariate regression analysis; the results showed that UICC stage, length_T, SUV_max_2, MTV1 and TLG2 were independent prognostic factors for OS (*p* < 0.05), with Wald values of 9.8,5.5,9.77,4.82 and 10.9 and hazard ratios of 2.7,2.1, 2,1.7 and 2.2, respectively (see detail in Table 3).

**Table 3:** Cox regression results

		UICC Stage	Length_T	SUV_max_2	MTV1	△MTV	TLG1	TLG2	△TLG
Wald		9.8	5.5	9.77	4.82	2.72	2.11	10.9	0.09
P valne		0.002	0.019	0.002	0.028	0.099	0.146	0.010	0.770
HR		2.7	2.1	2	1.7	1.53	0.648	2.2	0.9
95% CI	Upper limit	1.5	1.13	1.3	1.1	0.9	0.36	1.4	0.44
	Lower limit	5.2	3.9	3.1	2.8	2.5	1.16	3.5	1.84

*HR* Hazard ratio, *CI* Confidence interval

## Discussion

Radiotherapy and chemotherapy are important treatment approaches for EC [21]. For non-surgical EC treatment, radiotherapy with concurrent chemotherapy is the treatment of choice. Anatomical information from conventional barium swallow tests or CT cannot reflect the burden of metabolic active tumor volume. 18F-FDG PET/CT functional imaging was shown to be superior to conventional imaging for EC staging [22, 23]. A growing body of evidence shows that 18F-FDG PET/CT is a valuable tool with independent prognostic information for EC patients [24, 25]. However, studies about sequential 18F-FDG PET/CT in predicting (chemo-)radiation outcomes for EC are relatively sparse, especially in China. This study shows that TLG and its percentage change during radiotherapy have prognostic value regarding OS of EC patients. The same holds true for MTV. However, in our study baseline SUV_max_ did not have any prognostiv value. Interim SUVmax2 after about 40Gy of radiotherapy was at the other hand significantly associated with OS. These results suggest that in terms of prognostic stability, TLG and MTV seem to be quite robust in this cohort of patients.

SUV_max_ is commonly used as a prognostic parameter in clinical practice as it is easy to measure. However, SUV_max_ is affected by acquisition intervals, blood glucose, and insulin levels. In addition, SUV_max_ focuses on a single voxel with the highest FDG uptake, and thus cannot be used to evaluate the tumor’s overall metabolic state. As a result, researchers continue to debate the prognostic value of pretreatment SUV_max_ [12–15]. That may be the reason why our study showed that baseline SUV_max_1 was not associated with OS. SUV_max_2 was significantly associated with OS. This could be because SUV_max_ during treatment reflects the maximum metabolic activity of tumor tissue after antitumor treatment, e.g. response to therapy. Thus, a high value indicates high metabolic activity after conventional radiotherapy and chemotherapy. This non-response seems to be associated with high risk of local relapse and distant metastasis, and therefore with a worse prognosis. This finding is in line with one study that investigated interim PET and pathological examination of EC specimens’ response after neoadjuvant therapy [26].

MTV is determined based on a SUV threshold and ideally should reflect metabolically active tumor cells with increased glucose metabolism; thus, it reflects the overall metabolic state of the tumor. Several studies showed that MTV is a better prognostic parameter than SUV_max_ [20, 27–29]. This study showed that both baseline MTV1 and ΔMTV were correlated with OS. ROC curve analysis showed that the optimal prognostic threshold for OS was10.5 mL for MTV1. The published MTV thresholds varied across studies; nevertheless, low baseline MTV indicates a low tumor burden and is associated with a more favorable prognosis. Theoretically, MTV during radiotherapy reflects the volume of radiotherapy-resistant tumor cells; however, this study was not able to show that MTV2 was associated with OS. This may be because some patients had radiation induced inflammation in the tissue around the esophageal tumor after radiotherapy and chemotherapy, which interfered with the MTV measurement. As a result, MTV2 may not be able to properly reflect the actual interim-PET MTV, which was consistent with the findings from Erasmus et al. [16], Bar-Ad et al. [17], and Yuan et al [18]

TLG has recently become an emerging new prognostic 18F-FDG PET/CT parameter. TLG is the product of MTV and SUV mean. Studies have shown that TLG is non-inferior [30–32] and may even be superior to MTV [19, 20, 33] as a predictor, probably because it reflects both tumor metabolic activity and tumor volume. This study showed that all three TLG parameters, TLG1, TLG2, and ΔTLG, were related to OS (*p* < 0.05), suggesting that TLG is more robust and reliably to predict the outcome of radical (chemo-)radiotherapy in EC. However upon multivariate regression testing TLG1 and ΔTLG did not remain independent prognostic factors, which might however be due to the correlation with MTV and other PET parameters.

At present, SUV_max_, MTV, and TLG are commonly used PET/CT parameters to predict tumor response in interim PET. This study showed that TLG was a reliable sequential 18F-FDG PET/CT parameter for predicting outcome after radical (chemo-)radiation for EC. TLG could be less prone to the shortcomings of SUV_max_ and MTV discussed above. However it is important to mention that the calculation of TLG is strongly dependent on tumor delineation. Therefore also TLG has several limitations, even more when used during interim PET (TLG2 or ΔTLG) since there is no consensus for optimal delineation of FDG metabolic tumors in the course of radiotherapy. Simultaneously, like MTV, TLG also has the drawbacks of being influenced by inflammation induced by (Chemo-)radiation. That may be another reason why the Cox multi-factor regression did not show positive results for ΔTLG in our study. Non-tumour-affected oesophagus (NTO) on restaging PET may be a promising method to deal with the treatment-induced inflammation [34]. Additional PET biomarkers are under investigation for EC right now. Recent studies [35, 36] showed that texture analysis may become a promising new method for predicting EC prognosis; however, texture analysis is difficult to perform in clinical practice, and the results vary greatly across software packages. Furthermore, new tracers may minimize interference from radiation inflammation on PET/CT images [37].

The results of our research show that interim PET after 40 Gy of radiotherapy seems to be a promising tool to comprehensively evaluate the treatment response with reference to the baseline parameters. SUV_max_2 and TLG2 seem to be useful parameters to identify patients with high risk of recurrence. Patients with adverse prognostic factors may receive re-planning and higher dose to the region of high FDG uptake or additional therapeutic escalation to improve treatment outcomes or even might undergo surgery.

This study has some limitations. First, no official MTV threshold has been established, and we used the volume of the area greater than 40% SUV_max_, which requires further validation. Second, the heterogeneity in concurrent chemotherapy regimens may affect the results. Third, results can be prone to bias, inherent to any retrospective evaluation and should be interpreted as hypothesis generating.

## Conclusion

The results of our study indicate that sequential 18F-FDG PET/CT metabolic parameters have a good prognostic value for OS of squamous EC. It is recommended to repeat 18F-FDG PET/CT scan during chemoradiotherapy to improve clinical decision-making and individualize treatment.

## Abbreviations

CI: Confidence interval.
CT: Computed tomography
CTV: Clinical target volume
EC: Esophageal cancer
FDG: Fluorodeoxyglucose
GTV: Gross tumor volume
Gy: Gray
HR: Hazard ratio
ICRU: International Commission on Radiation Units and Measurements
KPS: Karnofsky performance status
m: Months
MTV: Metabolic tumor volume
NTO: Non-tumour-affected oesophagus
OS: Overall survival
PET: Positron emission tomography
PTV: Planning target volume
ROC: Receiver operating characteristic curve
ROI: Region of interest
SUV: Standard uptake value
TLG: Total lesion glycolysis
